# Developing mHealth IT for Older Adult Medication Safety: Remote Participatory Co-Design Using the RAPID Method

**DOI:** 10.2196/82366

**Published:** 2026-02-27

**Authors:** Jordan R Hill, Aaron Ganci, Noll L Campbell, Andrew C Pickett, Michelle A Chui, Ephrem Abebe, Richard J Holden

**Affiliations:** 1 Department of Health & Wellness Design, School of Public Health - Bloomington, Indiana University, 1025 E 7th St, Bloomington, IN, 47405, United States, 1 812-856-5032; 2 Department of Visual Communication Design, Herron School of Art and Design, Indiana University, Indianapolis, IN, United States; 3 Department of Pharmacy Practice, College of Pharmacy, Purdue University, West Lafayette, IN, United States; 4 Indiana University Center for Aging Research, Regenstrief Institute, Indianapolis, IN, United States; 5 School of Nursing, University of Wisconsin - Madison, Madison, WI, United States

**Keywords:** co-design, participatory design, remote, health intervention, older adults, digital health, pharmacy

## Abstract

**Background:**

Participatory co-design is a design approach that involves end users in intervention design and its use in health care applications has become widespread. Traditionally, co-design has been conducted in person in a laboratory-based setting; however, it has recently shifted to being performed remotely. Remote co-design has the potential to overcome some of the limitations of traditional in-person approaches, including expanding a study’s geographic reach, recruiting participants from underrepresented groups, reducing power imbalances between researchers and participants, and enhancing engagement through online tools. Given these benefits, further reporting and refinement of remote co-design methods are needed.

**Objective:**

This paper’s objective is to present our Remote and Accessible Participatory Intervention Design (RAPID) method and discuss the choices and challenges we encountered adapting participatory co-design for remote use.

**Methods:**

We adapted our previously developed 5-step in-person participatory co-design method for health intervention design. To apply the adapted co-design method, we recruited 2 groups of 5 participants (one of older adult pharmacy patients and the other of pharmacy staff) to design a digital kiosk for use by older adults to promote safe over-the-counter medication purchases in retail pharmacies.

**Results:**

Adaptations made to the co-design process were classified under the following categories: facilitation; collaboration, communication, and sensemaking; accessibility and universality; tangible tools and games; and research compliance. Anecdotally, the remote co-design process took longer when compared to in person due to shorter sessions and between-session refinement, but it allowed for flexible scheduling and makeup sessions when required.

**Conclusions:**

Our RAPID method offers an approach to remote co-design that other teams can implement or adapt to their needs. Our experiences with RAPID identify certain drawbacks to remote co-design; however, these are balanced by advantages in convenience and flexibility.

## Introduction

Centering patients in health intervention research has become a priority to ensure that interventions align with the patients’ needs, values, and priorities [1-6]. Patient-centered interventions are more likely to be adopted and implemented, maximizing their impact [6,7], and there is evidence that they result in improved health outcomes [8]. Participatory co-design methods are a formal way to involve intervention end users in product or service design to directly center user needs (eg, usability, acceptability, and usefulness) [9] including those of patients to design patient-centered interventions [1]. With the rise of the “digital health citizen” [10-12]— empowered patients who are active consumers and generators of their own health data—the demand for patient-centered tools and services will only increase. Co-design with patients and informal caregivers has been increasingly adopted in healthcare systems design to address these demands [13].

Traditional participatory co-design is performed face-to-face, requiring end users to physically travel to a secondary location to attend activities such as design workshops. Remote approaches to co-design had been considered before but became necessary during the COVID-19 pandemic [14]. Since then, remote co-design has been performed with various populations of interest including children [14-16], racially underrepresented young adults [17], and caregivers of patients with dementia [18,19]. The applications have been similarly heterogeneous from supporting antibullying [16] to helping family caregivers with legal and financial planning [18,19].

While there are challenges to implementing participatory co-design (eg, differences in familiarity with technology and difficulty aligning goals and language of various stakeholders) [20], remote co-design may have advantages over traditional face-to-face approaches. Pioneers of remote co-design methods report recruiting participants from wider geographic areas [15,21] and extending their study’s reach to populations sometimes excluded from research [15,17]. The remote format can also facilitate certain user interactions. Bertran et al [21] described how study facilitators and designers were able to disappear “behind the scenes” to attain a more naturalistic perspective of participants interacting with their prototype. Similarly, Fails et al [14] reported that the lack of researchers’ physical presence balanced some of the child-adult or facilitator-participant power dynamics. Additionally, some online features such as chats during video calls enabled more participants to engage, respond, and discuss [17]. Some remote co-design methods (eg, surveys and asynchronous feedback) also enable the collection of large amounts of data when compared to small studies conducted in person [22]; this is especially important when considering the big data implications of widespread digital health use and data generation. With the benefits becoming increasingly apparent, it is imperative to report on and improve upon the use of remote participatory co-design methods.

This paper’s objective is to present our Remote and Accessible Participatory Intervention Design (RAPID) method. There is a clear need to develop RAPID because, while there have been some methods-focused or instructional papers on remote co-design [14,23,24], we have not found any that specifically focus on engaging older adults in remote co-design. RAPID addresses this gap as it was adapted specifically for older adults and has been used successfully with older adults in other studies [18,19]. There are challenges in engaging older adults in digital research: the digital divide persists with fewer older adults using the internet [25], having access to broadband internet at home [26], and owning a smartphone [27] when compared to other demographics; and older adults are stereotyped as having less technological ability than younger adults [28]. However, older adult technology adoption is growing [27,29]. Nearly 1 in 5 Americans is over the age of 65 years (18%) [30], and this age group experiences more chronic diseases. It is important that digital health interventions are designed centering older adults as they are likely to be widespread users in the future.

In this paper, we discuss the choices and challenges we encountered in adapting participatory co-design for remote use. Building on existing work, we organize our results using the framework outlined in Mallakin et al [23]. We hope this method will be useful for others designing patient-centered interventions for older adults and potentially other populations who may experience challenges with co-design, and that it will encourage further involvement of older adults in digital health intervention design.

## Methods

### Overview

The RAPID method was originally developed for and applied in a specific study [31]. However, it was also envisioned as a formative method that could be adopted for future studies, further observed in practice, refined, and adapted over time. The initial study, called Senior Station, had the goals of co-designing and testing a technological intervention (a kiosk) to support older adults in making safer over-the-counter (OTC) medication decisions by providing them with information on which medications had ingredients that may not be safe for their brain health. More detail on the intervention designed can be found in a separate publication [31].

**Figure 1.**
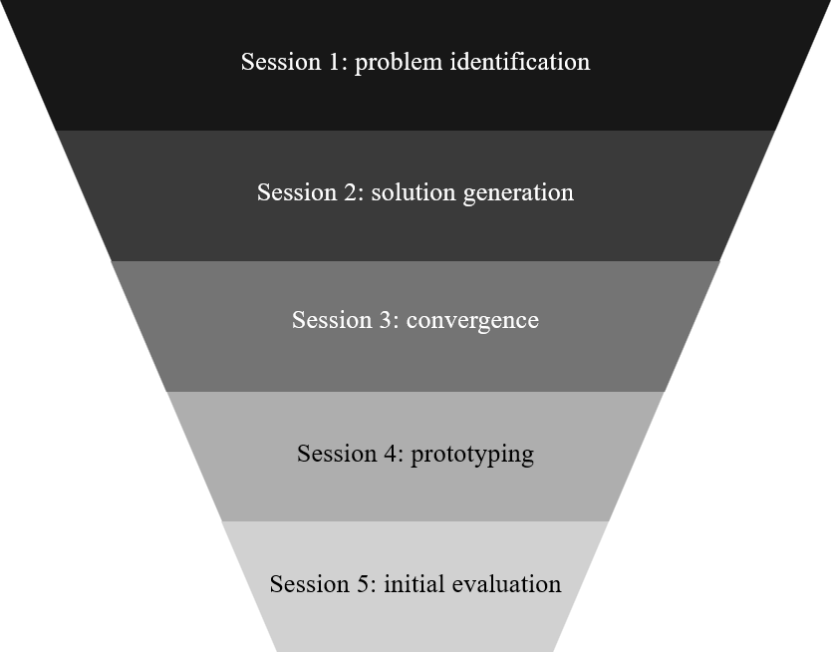
Remote and Accessible Participatory Intervention Design (RAPID) co-design stages.

To develop RAPID, we adapted our previously developed 5-step in-person participatory co-design method (Figure 1) for remote delivery. This funnel framework was developed for health intervention design in an older adult OTC medication decision-making study [32] and subsequently applied in a broad array of projects and settings [18,19,31,33]. Details on each RAPID session and how we adapted them for remote settings are described in the results section.

To apply RAPID, we recruited 2 groups of 5 participants each (N=10): one of older adult pharmacy patients and the other of pharmacy staff. All participants were recruited from a large pharmacy chain retailer in the United States.

Older adults, aged 60 years or older, were those who had previously purchased or planned to purchase OTC medication, could communicate in English, and were able to join a video call from a computer, tablet, or smartphone. Potential participants were approached in person by a member of the research team at the pharmacy retailer and were screened for eligibility if they expressed interest in participating. The research team member reviewed a paper study information sheet and obtained verbal consent. Participants provided their contact information for future coordination to attend the remote design sessions.

Pharmacy staff were pharmacists and technicians who had previous experience counseling older adults on the purchase of OTC medications, could communicate in English, and were willing and able to join a video call. They were recruited via the research team’s professional contacts at the same retailer from which older adults were recruited and filled out an online survey with their contact information if they were interested in participating. A member of the research team then contacted pharmacy staff, reviewed the consent form, obtained verbal consent for participation, and captured demographic information via a Qualtrics survey.

Each session was facilitated by 3 to 4 members of the research team. An expert in participatory co-design facilitation acted as “master of ceremonies.” The “master of ceremonies” was responsible for leading the agenda, guiding the participants through co-design activities, and prompting participants to assess each other’s design ideas. In later sessions, the same individual guided participants to review solution prototypes. The second role in each session was an administrator or “problem solver.” They ensured participants had the information and resources they needed to participate in the study. This role was critical to help some participants troubleshoot technical issues during the session. The third role was a human-computer interaction expert who observed participants’ design ideas through the lens of implementation and usability. Their role during the discussion was mainly asking clarifying questions. Finally, a scribe participated in the sessions to capture participant ideas in real time. In our study, this person was a design student who was also able to sketch prototypes in real time.

The 5 steps of the RAPID method were adapted for remote delivery as shown in Table 1.

The observations in the following sections are based on team self-reflections and a review of notes, correspondence, and other documents over the course of the project. There was no systematic method in place for the analysis of the results of the RAPID sessions.

**Table 1.** Remote and Accessible Participatory Intervention Design (RAPID) procedure for remote co-design.

Step	Procedure	Remote approach
Problem identification	Introduce the research team and have participants introduce themselves. Have participants describe their experiences with the challenge or situation of interest (eg, tell a story) Visually represent these stories using journey maps to identify pain points and targets for intervention Explicitly introduce the concept or challenge the design seeks to address Present different choices or scenarios to participants to determine what factors are most important (eg, do cost savings outweigh warnings of risks) and which design features are more desirable	Videoconferencing (Zoom; Zoom Communications Inc) Microsoft PowerPoint presentation on shared screen Turn-taking Heavy facilitation and experienced facilitator
Solution generation	Present a problem scenario to participants with an undescribed solution to help them Guide participants through brainstorming the design of different aspects of the solution, such as size, information communicated, advertisements, features, and symbols. Have participants roughly sketch their designs on whiteboards and present them to the group. This enables participants to express themselves creatively, provide diverse perspectives, and turn abstract ideas into something tangible. Take photos or some other record of the drawings	Videoconferencing (Zoom) PowerPoint presentation on shared screen Turn-taking Heavy facilitation and experienced facilitator Whiteboards mailed to participants before the session Photos of drawn designs taken over Zoom or by participants and emailed to the research team
Convergence	Present low-fidelity, hand-drawn designs to participants based on ideas in previous session. Low-fidelity prototypes make it clearer to participants that design ideas are preliminary and not “set in stone.” Walk through the sequence of use of design, seeking feedback on each design and making it clear that designs were flexible. Ask participants to provide more details (eg, specific information and what is prioritized) on designs. The goal is to converge the multiple designs into one with which to proceed.	Videoconferencing (Zoom) PowerPoint presentation on shared screen Turn-taking Heavy facilitation and experienced facilitator Hand-drawn designs on a tablet or computer and shared on screen during session
Prototyping	Present a higher-fidelity prototype (eg, a clickable wireframe) that is the result of feedback from the previous session. The higher-fidelity prototype made the design feel more real and indicative of the final intervention. Solicit feedback from participants	Videoconferencing (Zoom) PowerPoint presentation on shared screen Turn taking Heavy facilitation and experienced facilitator Prototype in Figma (Figma, Inc.) and presented on screen during session
Initial evaluation	Present the final design based on the design sessions from both participant groups Solicit feedback from participants	Videoconferencing (Zoom) PowerPoint presentation on shared screen Turn taking Heavy facilitation and experienced facilitator Prototype in Figma and presented on screen during session

### Ethical Considerations

This study was approved by the Indiana University Human Research Protection Program (institutional review board number 13200) and co-design sessions occurred from January 2022 to November 2022. Participants were compensated US $50 in gift cards for each session they attended in addition to bonus payments as incentives to avoid participant attrition: US $100 bonus for attending all of the first 3 sessions or US $250 bonus to attend all 5 sessions (for a potential total of US $500 in compensation). A research team member reviewed a paper study information sheet with participants and obtained verbal consent. All participants were able to opt out of the study and terminate their participation at any time. Any transcripts and meeting notes generated during sessions were de-identified to protect confidentiality and privacy. Session photos of participant designs or sketches did not contain any identifiable information.

## Results

Here we describe our adaptations, organized retrospectively using Mallakin et al’s [23] framework for considerations for remote co-design.

### Facilitation

#### Definition

Facilitation is the process by which designers and researchers “set the scene,” then guide participants through the design process to stimulate creativity and generate collaborative design solutions.

#### Remote Considerations

Remote facilitators may not be aware of the physical space or equipment limitations or assets of the remote co-designers. They cannot directly set up, demonstrate, or manipulate the co-designers’ physical design space or equipment and are more constrained in their strategies to establish trust, open dialogue, or rapport such as one-on-one time, informal side conversations, or physical gestures. Remote sessions are typically shorter than in person, which are often day-long or half-day events.

Successful co-design activities rely on open conversation where participants generate prototypes, pitch ideas, provide feedback, and then build on one another’s concepts. When online, this process is impeded because the ability to present ideas is limited and the nature of discussions is restricted. For example, it is hard to manage crosstalk during online discussions, which results in participants being more hesitant to contribute out of fear of interrupting another person.

While in-person sessions feel more like a collaborative group conversation, the online session requires significantly more facilitation. For example, in an in-person setting, it would be easy to look at 2 participant prototypes at the same time to compare them. That is much harder online and would require the facilitator to collect images of the prototypes before talking about them.

#### Our Approach

The co-design facilitator had extensive experience with in-person facilitation and anticipated what interactions could be replicated remotely versus required accommodation. The entire research team, including the facilitator, met extensively before the start of co-design activities to fully conceptualize the design problem and develop cases, scenarios, and prompts to guide participants through the design process. More time was allotted in the first session for rapport building with the research team and among the participants, using turn-taking to ensure everyone had a chance to speak. The facilitator was intentional to lead discussion and call on participants, as a countermeasure to the awkwardness of interrupting a speaker in an online format (due to audio delays, the abruptness of teleconference interruptions, and uncertainty about the source of interruption). Prototyping tools conducive to remote co-design were selected (eg, Adobe XD, Figma, and individual whiteboards). To facilitate co-designers' critiquing of each other, the facilitator guided participants to offer initial feedback then build on each other’s initial ideas rather than directly grading designs.

#### Lessons Learned

The facilitator is important in remote settings because they must be skilled in co-design methods and adjust those methods to suit the setting. Facilitators cannot assume methods that work in person will be as effective online. They should also have strong technology skills for more seamless remote co-design meetings.

Remote settings can feel socially distant, impersonal, or more businesslike compared to in person. A facilitator needs to be intentional about creating a creative and open environment where participants feel comfortable working together. This could include focusing on friendliness by adding a joke to the session or asking pointed questions to get participants to talk about something they mentioned in passing.

Online meeting conventions and limitations (eg, one person speaking at a time, no cross-talk, and limited body language cues) can hamper study interactions. It often falls on the facilitator to smooth the conversation and facilitate group communication.

Facilitator expectations regarding how many design activities can be accomplished in each session need to be adjusted when performing co-design online. There are 2 factors of online co-design that impact a facilitator’s time management. First, it is harder to hold participants’ attention, thus favoring shorter sessions. Second, technical issues will arise in online sessions. Our team lost time during most sessions waiting for participants to join, troubleshooting technology issues, or clarifying instructions.

### Collaboration, Communication, and Sensemaking

#### Definition

Collaboration, communication, and sensemaking are the processes by which participants work together with the research team to produce co-design.

#### Remote Considerations

Participants are not co-located in the same physical space (eg, a design studio) and must meet and interact virtually. There is no shared physical whiteboard, flip chart, or other materials to which every participant has real-time access to externalize their thoughts in a manner directly visible to others. Some designs, for example, those that are drawn or constructed on nondigital artifacts (eg, scrap paper) or have 3D elements (eg, gestures), may not be easily shared on a screen or will need to be digitized. Differences in familiarity and comfort with remote technologies may affect how individuals engage in collaboration, communication, and sensemaking.

#### Our Approach

To host online sessions, we selected Zoom videoconferencing software (Zoom Communications Inc) as it was freely accessible on a range of devices and operating systems, widely used during the pandemic, supported through the research team’s institutions, and supported recording for later review and analysis. A design student attended our virtual sessions to take notes so that the facilitator could focus on supporting interaction.

**Figure 2.**
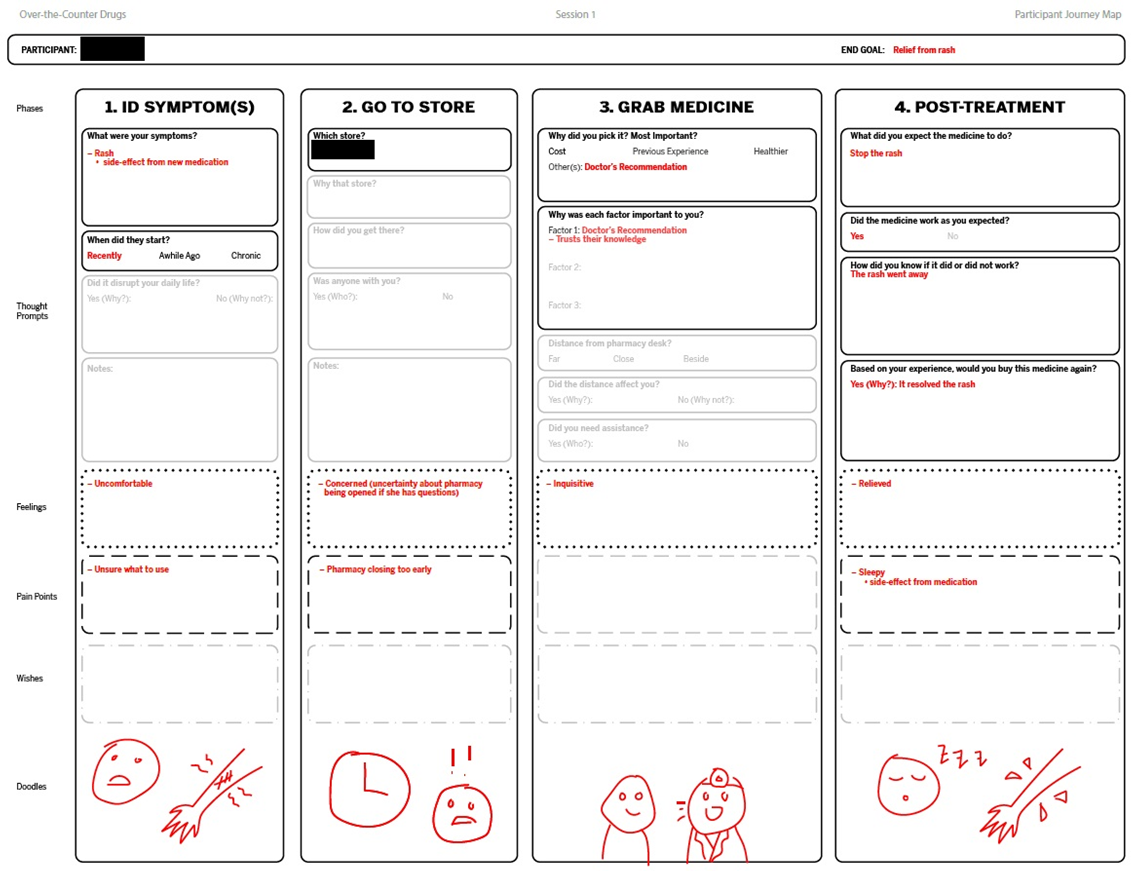
Deidentified journey map notes.

**Figure 3.**
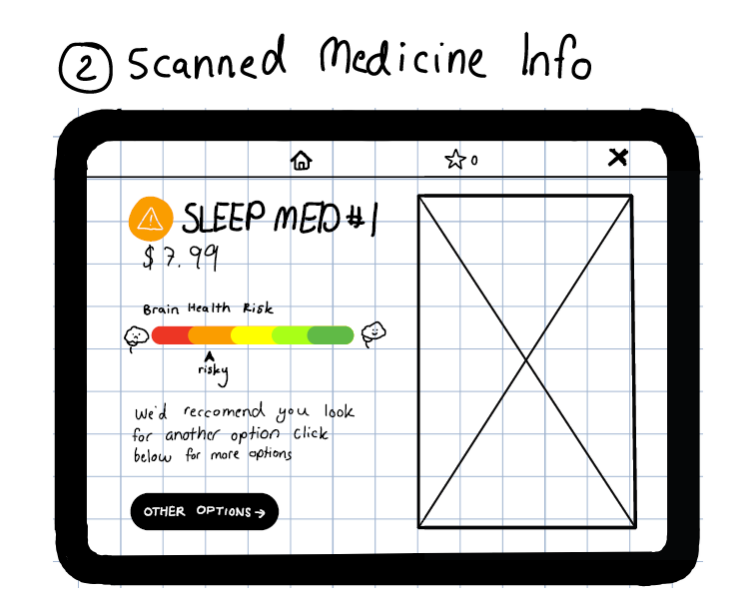
Low-fidelity wireframe.

A journey map template was created to help the designer capture notes in a manner that could be easily shared with others (Figure 2). Our designer created simple, low-fidelity wireframes summarizing the designs that could be easily shared online, but despite being digital, looked rudimentary enough to facilitate critiques (Figure 3).

#### Lessons Learned

Conversation does not flow as naturally or collectively online as in person, so participants are disinclined to build on each other’s ideas, implying they may perceive their roles as developing individual designs rather than a single co-owned design. Participants were frequently distracted in the online environment (phone calls, family members in the home, and so on) and felt more comfortable attending to distractions and multitasking.

The primary physical medium for online co-design, a 2D computer screen, is not as inherently a creative space as a 3D design studio. An atmosphere of creativity is therefore often created through the activities, words, and interactions of co-design rather than the setting. Physical tools and virtual environments could be used to increase creativity.

Participants did nevertheless develop a sense of community and comfort over the course of 5 sessions, despite being in a relatively sterile remote setting.

### Accessibility and Universality

#### Definition

Accessibility and universality refer to the degree to which a wide and inclusive range of individuals can and do participate in co-design.

#### Remote Considerations

Comfort with technology and access to a stable internet connection are important considerations for remote co-design, especially with certain groups such as older adults. Unexpected problems (eg, technical difficulties) are more likely to occur in remote settings, and not every participant is equally adept at using online interaction features such as typing private or public messages, digital gestures (eg, raised hand) and reactions, electronic whiteboard, screen sharing, audio sharing, muting or unmuting, and hyperlinking. Communication can be hampered or interrupted by having to concurrently deal with technology issues such as typing, audio, video, switching windows, computer slowness, and internet connectivity. Not all participants joined from a desktop or laptop computer. Those who joined using cellphones were hampered by having smaller screens.

#### Our Approach

One member of the research team spent significant time outside of design sessions working with the participants, including the older adults, to ensure they could join a videoconference and practicing with them to increase comfort. In one case, this required live phone assistance prior to every session. We designated a “problem solver” to provide support for emergent accessibility needs such as troubleshooting technical difficulties and phoning participants who did not join the session on time. To compensate for participants joining from smaller screens (eg, cellphone), the facilitator spent time during the session explaining what was being shown on screen.

#### Lessons Learned

Including participants who were less comfortable with technology was a benefit, not a downside, of the remote co-design approach, as they provided a novel perspective that was especially important in our attempts to design universally usable technology for older adults. The problem solver was invaluable to the smooth operation of design sessions as they handled unexpected problems without disrupting the session for the facilitator, designer, and participants. The provision of technical support during and outside of sessions requires additional personnel and associated expenses.

### Tangible Tools and Games

#### Definition

Tangible tools and games refer to the tools and games used to encourage participants’ creativity and facilitate co-design work.

#### Remote Considerations

Traditionally, co-design tools and games are hands-on and require physical items. Games are often experienced in a playful, informal, low-risk context, whereas online meetings may be associated with formal, serious, and more sterile functions (eg, work).

#### Our Approach

Before each session, participants were mailed relevant materials for that session. This included prototyping tools or prototype concepts for their review. We provided personal whiteboards and markers to participants, mailed directly to their homes by an online retailer. Some participants spontaneously did “homework” to create preliminary designs before the session, thus extending creativity beyond the time and space confines of the formal design sessions.

#### Lessons Learned

Not all traditional co-design tools and games could be translated to the online setting, and some could not be used. New or adapted tools need to be intentionally and innovatively planned to enable activities such as online sharing of participant-created prototypes. It is more difficult to adjust or introduce new tools or ideas in real-time online, and modifications to set schedules are often not well received or understood in online spaces. As such, remote sessions require additional planning and forethought. The spirit of fun and creativity of in-person games and activities felt constrained in online encounters. Our tools were limited to the software and hardware we chose to use, making it difficult or impossible to introduce ad hoc tools and activities often available in a traditional design studio.

Mailing whiteboards was a low-cost, low-tech option that provided participants the ability to do some design activities without a steep learning curve. Participants were easily able to photograph their whiteboard designs and send them to the research team. Other options were dismissed as too costly, complex to learn, or disruptive to the project timeline.

**Figure 4.**
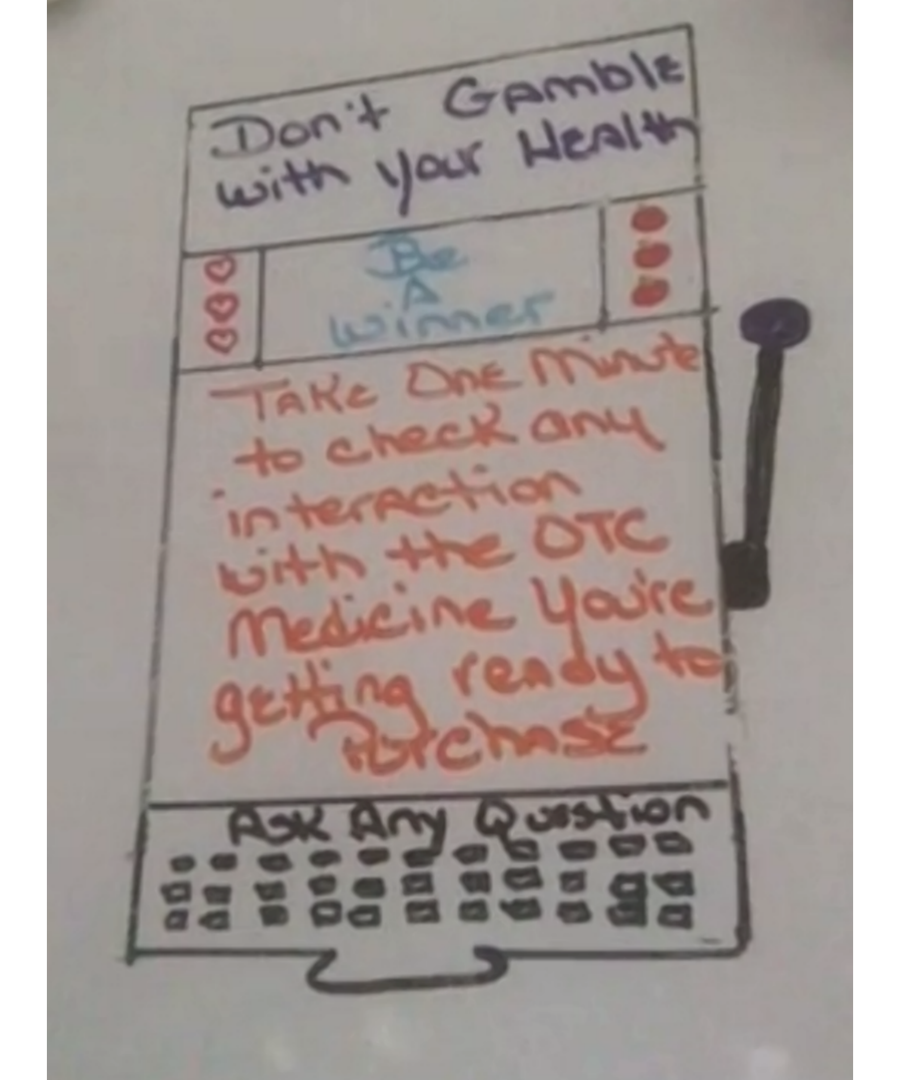
Participant design on whiteboard.

Participants doing extra work between sessions produced creative designs (Figure 4) and may have increased reflection. It is possible in our project and in others that working between sessions would allow participants to engage others in their household, social network, or community to enrich their designs. In other cases, working independently could allow co-designers the ability to use novel tools such as generative artificial intelligence that could stimulate their creativity and self-efficacy.

### Research Compliance

#### Definition

Research compliance is the degree to which co-design adheres to the applicable ethical principles and requirements

#### Remote Considerations

When participants are in a public online gathering place, especially one that is being recorded, additional steps are needed to ensure privacy for participants and provide opportunities for participants to privately interact with researchers. While some physical affordances such as private rooms may have online analogs (eg, private breakout rooms), their use may not be as easy.

The physical setting for remote interactions may be less controllable for researchers and co-design participants who may be joining from a shared space such as their home, workplace, or coffee shop. This impacts both privacy and confidentiality considerations, particularly when using video or audio recording.

#### Our Approach

We conducted recruitment and informed consenting in person in community pharmacies, rather than during the online sessions, and provided older adult participants with printed study information sheets. Sessions were hosted using institutional videoconferencing accounts (Zoom) for added security. Standard web-based data security approaches applicable to all forms of internet-based research (eg, deidentification of data, and storage of data on secure, approved servers) were used.

#### Lessons Learned

Participants do join remote co-design sessions from locations such as workplaces, homes, and cars, and those joining from work often turned off their cameras, which made conversations more difficult.

### General Lessons

In addition to considerations inspired by the Mallakin et al [23] framework, implementing RAPID produced the following general takeaways.

Drawing on our prior experience with in-person co-design, we observed that the remote co-design process took considerably longer in the remote format. This is likely because RAPID sessions were in shorter 90-minute bursts, and in between sessions, designers spent considerable time refining the designs and preparing materials for the next round. This was advantageous for advancing the designed product but increased the total project duration.

A second general lesson is the need for flexibility and willingness to implement contingencies for the unexpected. For example, we recommend over-recruiting for each group to ensure an adequate number of participants. In both the older adult and pharmacy staff groups, we had fewer than 5 participants participate in all but 1 session. However, a benefit of RAPID was the ease of adding makeup sessions when multiple participants could not attend a prior session.

Third, we performed our remote co-design sequentially with the 2 groups due to recruitment and timing considerations. We believe that it would be beneficial to complete the sessions concurrently to ensure that each group’s designs are not incompatible with the other’s. For example, the older adult participants wanted a scale to demonstrate a medication’s effectiveness versus safety. Upon further investigation and discussion with pharmacy staff, such a scale was infeasible and would not apply to all users. Our research team made some decisions that were not purely end user driven to reconcile differences between the 2 groups’ designs.

### Participant Feedback

We did not explicitly or systematically collect feedback on the co-design sessions from participants. However, during the last session, participants provided unprompted opinions on their experiences. The feedback was positive and older adult participants expressed their enjoyment in participating:

*“This was fun! I enjoyed it.”*

[Female older adult participant, age 79]

*“Thanks for the opportunity to participate.”*

[Male older adult participant, age 74]

*“Oh my gosh, it was really really fun. I felt so important!”*

[Female older adult participant, age 66]

## Discussion

### Principal Findings

We produced RAPID by adapting our co-design method to be performed remotely and gained valuable learning to guide future remote co-design projects.

The primary lesson learned was that the online setting is intrinsically less creative and more socially distant than traditional, in-person environments. Our experiences of constraints on participant rapport and trust [14,15], engagement [14], and fewer generative side conversations [19] have been reported in other studies. Practically, this emphasizes the importance of having skilled and experienced facilitators (ie, facilitators with strong technology and communication skills, and an in-depth understanding of transforming user needs into design choices), using activities that promote social interaction among participants (eg, ice breakers, jokes, pointed questions, and turn-taking), and anticipating delays and distractions throughout sessions.

In line with other literature, we found that the remote co-design, compared to our past experiences with traditional in-person co-design, had higher participant attrition than expected [16,17,19], required more researcher flexibility [14,15,17], and was slower [14,16]. We attribute our slower process to shorter design sessions, additional between-session activities, and scheduling serial rather than concurrent independent co-design groups (older adult vs pharmacy staff). The restriction on session duration, chosen to manage participant fatigue, may require the most creativity to overcome.

Our experience with participant differences in technology comfort and experience validates recommendations by Jolliff et al [19] and Fails et al [14] to provide technical support outside sessions, during sessions, or both, as we did.

Despite certain disadvantages of remote co-design, there were also benefits that promoted the continued use and refinement of RAPID. The convenience for participants to participate in research from anywhere with a suitable internet connection is the greatest advantage of any remote research methodology and applies to remote co-design. We engaged participants who otherwise may not have participated due to geographical separation, weather, or work or family obligations. This benefit is noted by others [14,15]. We identified an additional financial benefit by avoiding transportation and facility costs.

We found that, without prompting, participants completed extra work between sessions (“homework”). This enhanced design creativity during sessions and comports with the general benefits of presession activities and time for reflection in co-design [15,17,19]. We recommend prospectively incorporating “homework” into future RAPID applications to help reflect and create without researcher influence or time pressure.

### Limitations

Our experiences, lessons learned, and recommendations were limited in several ways, including the fact that the study sample was small, and this was an early-stage, exploratory study. First, we took a pragmatic but unsystematic approach to creating and reflecting on RAPID, disproportionately leveraging our team’s expertise and adapting “on the fly.” This is to be expected with a method that arose rapidly out of necessity (ie, COVID-19 pandemic restrictions). Indeed, the ability to fail safely and learn quickly is a hallmark of strategies such as Agile [34,35]. However, with time and experience, new approaches such as RAPID and frameworks such as Mallakin et al [23] can be more systematically evaluated and refined. Second, we did not independently measure success of RAPID or compare it to traditional in-person co-design, a necessary future research direction. Explicitly soliciting participant feedback would also be an important future step in assessing the method. Third, despite efforts to include a sufficiently large and heterogeneous set of co-design participants, the first users of RAPID needed to own a computer, tablet, or smartphone with stable internet and sufficient technology comfort to participate in the study. Even if devices with internet access were provided, something we have done in other studies, this may exclude individuals who do not feel comfortable enough with technology.

### Conclusions

Remote co-design is increasingly used. Our RAPID method offers an approach to remote co-design that other teams can implement or adapt to their needs. Our experiences with RAPID identify certain drawbacks to remote co-design; however, these are balanced by advantages in convenience and flexibility. Our use of RAPID also offers various strategies to address challenges to remote co-design.

## Abbreviations:

OTC: over-the-counter
RAPID: Remote and Accessible Participatory Intervention Design
